# Exploring Indonesian midwives’ training experiences and knowledge related to cervical screening and HPV DNA testing in East Nusa Tenggara province

**DOI:** 10.1177/17455057261487155

**Published:** 2026-09-06

**Authors:** Sandra Olivia Frans, Ariane Utomo, Linda Rae Bennett

**Affiliations:** 1 Nossal Institute for Global Health, Melbourne School of Population and Global Health, 50066 University of Melbourne, Carlton, VIC, Australia; 2 School of Geography, Earth and Atmospheric Sciences, 549286 University of Melbourne, Carlton, VIC, Australia

**Keywords:** cervical cancer, cervical screening, HPV DNA test, Indonesia, midwives

## Abstract

**Background:**

Indonesia has the highest rates of cervical cancer mortality in Southeast Asia, mainly due to poor access to and low uptake of cervical screening. Indonesian midwives are primarily responsible for conducting cervical screening, yet there is very limited research on their relevant knowledge, training and capacity to provide screening.

**Objective:**

This study explored midwives’ experiences of cervical screening training and their knowledge related to CC, cervical screening using Visual Inspection with Acetic Acid (VIA), and Human Papillomavirus (HPV) DNA testing in East Nusa Tenggara, Indonesia.

**Design:**

A qualitative study design was employed, based on primary data collected through fieldwork using semi-structured interviews and supplemented by a knowledge assessment questionnaire.

**Methods:**

Twenty-four midwives across two districts, one semi-urban and one rural, participated. Qualitative data were analysed using a codebook thematic analysis approach, and questionnaire data were descriptively analysed.

**Results:**

Midwives’ mean knowledge score was 75%, with midwives trained in cervical screening scoring higher than untrained midwives. Our findings highlighted inequitable access to training, with midwives in the more urban district having more access to training and higher knowledge. Midwives emphasised that adequate training and continuous practice of the VIA were crucial to building confidence in service delivery. Key knowledge gaps included poor understanding of the relationships between HPV infection, pre-cancer lesions, cervical cancer and its prevention. Midwives’ poor knowledge of cervical cancer aetiology and asymptomatic pre-cancer lesions resulted in only recruited women who came with associated symptoms.

**Conclusions:**

Geographical inequality in human resource investment in remote areas limits women’s access to quality cervical screening and impedes progress towards cervical cancer elimination. Supporting midwives through thorough and up-to-date training, including HPV DNA testing, is essential to increase screening access and uptake, and for the transition to HPV DNA testing.

## Introduction

Cervical cancer is a preventable yet leading cause of cancer-related death among women in Low- and Middle- Income Countries (LMIC).^1,2^ In Indonesia, cervical cancer is the second most common cancer in women, with approximately 37,000 new cases detected and 20,708 deaths annually, the highest mortality rates in Southeast Asia.^3^ Three-quarters of these deaths are due to late detection, which is linked to limited access and low uptake of screening,^4,5^ with only 10% of Indonesian women ever undergoing cervical screening,^3^ similar to other LMIC contexts.^6,7^ WHO’s Global Strategy to Eliminate Cervical Cancer endorsed primary health care as the preferred entry point for CC prevention.^8^ Therefore, strengthening the health workforce, including midwives, at the public primary care level is crucial to achieving cervical cancer elimination. This is particularly relevant in countries such as Indonesia, with low health worker-to-population ratios and large proportions of the population who are dependent on public health care services.^9^

Indonesian midwives provide maternal and child health (MCH) services and are also designated as the primary providers of cervical screening under *Indonesia’s National Cervical Cancer Elimination Plan* 2023-2030.^10^ Cervical screening is currently conducted primarily using the Visual Inspection with Acetic Acid (VIA) technique, with plans to expand the availability of HPV DNA testing in the coming years. While government regulations stipulate that both trained medical doctors and midwives can provide cervical screening,^11^ studies across provinces in Indonesia show that midwives are responsible for providing the most significant portion of VIA services in the public sector.^12,13^

Cervical screening training improves paramedical professionals’ knowledge and skills.^14^ However, the relationship between knowledge, training and implementation is not always straightforward and varies across settings. A survey of health workers in Ethiopia revealed that while 95% agreed that cervical cancer is a severe disease and screening is crucial, only half reported adequate training to conduct screening.^15^ In remote Cameroon, only one-third of the 200 health professionals were aware of the cause of cervical cancer; as a result, just 15% of them recommended cervical screening.^16^ Moreover, much of the existing literature on cervical screening focuses on barriers to women’s access to screening,^17–19^ with comparatively less attention on health workers’ training and applied skills. In Indonesia, despite the importance of midwives’ role in cervical screening, only one peer-reviewed publication has investigated their screening capacity, which reported inconsistent levels of midwives’ knowledge of cervical screening and inadequate training for midwives related to establishing competency in the interpretation of VIA results.^20^

Geographical inequities further affect midwives’ professional development and working conditions.^21,22^ East Nusa Tenggara (NTT) province, located in eastern Indonesia, has significantly poorer sexual and reproductive health outcome indicators compared with national-level indicators, such as higher unmet need for family planning (17.6% vs 10.6%), and a higher maternal mortality rate (316/100,000 vs 189/100,000).^23^ Midwives in rural NTT have a broad responsibility beyond their professional capabilities, despite having no additional training.^24^ Addressing the gap in research with Indonesian midwives, this article explores their training experiences related to cervical cancer screening and their knowledge related to screening and Human Papillomavirus (HPV) DNA testing in two districts in NTT province. The findings can inform the development of more tailored and comprehensive training for health workers, which will indirectly contribute to the elimination of cervical cancer.

## Methods

A qualitative investigation was employed, drawing on primary data collected through fieldwork using semi-structured interviews and supplemented by a knowledge assessment questionnaire to support the interpretation of findings in the context of limited resources.^25^

This article seeks to answer three key research questions:

• How do midwives’ experiences of cervical screening training differ between semi-urban and rural settings?
• What is the knowledge of midwives related to cervical screening and HPV DNA testing?
• What is the relationship between training experiences and knowledge of cervical cancer prevention among midwives?

### Settings

This study was conducted in NTT, an eastern province of Indonesia, chosen because of the low rates of cervical screening reported in this area in 2023,^26^ and because this province is underrepresented in women’s health research due to remoteness. The study was undertaken in two districts in NTT: Kupang Municipality, the capital of the province, representing a semi-urban setting and *Timor Tengah Selatan* (South Middle Timor *-* TTS), representing a rural setting. The districts were purposively selected to compare the geographical area and the first author’s prior experience and familiarity with the local context.

Public primary health services in Indonesia are delivered through *Puskesmas* (*Pusat Kesehatan Masyarakat* – Public Health Center, PHC) at the sub-district level. Each PHC is supported by auxiliary PHCs and smaller village health posts in remote communities, which are typically staffed by a midwife and/or a nurse.^27^ PHCs vary widely in the size of the population they serve, with communities ranging between 10,000 and 50,000. PHCs in the research are also varied in terms of the number of staff available, but the key staff roles typically found in PHCs include medical doctors, midwives, and nurses.

Until recently, cervical cancer prevention in Indonesia relied on secondary prevention through VIA screening. The national HPV vaccination program was introduced in 2023, while HPV DNA testing began phased implementation through pilot programs in 2024. In NTT, women with a positive VIA result were typically managed with cryotherapy in cases when this service and a trained physician were available; alternatively, they were referred to another facility for further assessment and management.

#### Sampling and recruitment

This paper forms part of the first author’s doctoral research exploring midwives’ experiences of delivering cervical cancer screening services in NTT, using a health systems perspective. In addition to qualitative interviews, a facility survey assessing readiness for cervical screening was also conducted; findings from the facility survey will be reported elsewhere. PHCs were purposively selected in consultation with the District Health Office (DHO) in Kupang and TTS districts. Selection was based on geographical location and service volume to ensure variation in service delivery contexts. At the time of the study, Kupang district had 11 PHCs across six sub-districts, while TTS had 36 PHCs across 32 sub-districts. Four PHCs in Kupang and eight PHCs in TTS were invited to participate through formal recruitment letters and participant information statements; all agreed to participate.

Midwives were recruited using purposive criterion sampling. We sought to recruit two categories of midwives from each participating PHC: (1) the midwife responsible for coordinating cervical screening services (VIA coordinator) and (2) a midwife who had been involved in community outreach activities related to cervical screening. Eligible participants were required to have worked at the PHC for at least one year to ensure familiarity with the local service context. No additional exclusion criteria were applied. Information about the study was distributed through the participating PHCs, and eligible midwives who expressed interest were contacted by the field researcher. In nine PHCs, the VIA coordinator was interviewed first and subsequently assisted in identifying another eligible midwife who met the second sampling criteria. In the remaining three PHCs, both participants were recruited concurrently. In total, 24 midwives participated in the study, including seven from four PHCs in Kupang and 17 from eight PHCs in TTS.

### Data collection

Data collection was conducted between January and April 2025. Prior to the interviews, all participants completed a self-administered questionnaire assessing their knowledge of human papillomavirus (HPV), cervical cancer, cervical screening and HPV vaccination. The questionnaire consisted of 14 true-or-false statements and was administered in person using paper-based forms. Nine items were adapted from previous studies,^28,29^ while the remaining items were developed based on a review of Indonesian national guidelines and WHO recommendations to ensure relevance to current cervical cancer prevention practices. To establish context validity, the questionnaire was reviewed by technical staff from the Indonesian Ministry of Health with expertise in cervical cancer prevention programs. The questionnaire was pilot tested with three Indonesian midwives who were not affiliated with the study sites to assess clarity, comprehension, and relevance. All participants completed the paper-based questionnaire independently. The questionnaire items and the proportion of correct responses for each item are presented in Table 2.

Following questionnaire completion, qualitative data were collected through semi-structured, face-to-face interviews. The interviews explored midwives’ experiences of implementing cervical screening and how these responsibilities were integrated within their broader workload. Topics included participants’ professional roles, cervical cancer prevention activities within their PHCs, screening provision and follow-up processes, perceived barriers and facilitators to screening implementation, and training experiences related to cervical screening (S2 File).

The interview guide was developed collaboratively by all authors. Interviews were conducted by the first author, a female medical doctor from TTS with previous clinical experience in a PHC in the district. All interviews were conducted primarily in Indonesian; however, participants occasionally used local dialects commonly spoken in the area. Most interviews were conducted in a private room at the PHC, while two interviews took place in a local restaurant according to the participants’ preference. Interviews were audio-recorded with participants’ permission and lasted between 31 and 76 minutes (mean: 44 minutes). Qualitative methods and reporting were guided by the Consolidated Criteria for Reporting Qualitative Research (COREQ) guidelines^30^ (S1 File). After seven interviews in Kupang, the authors met online to discuss the emerging findings. This process was repeated midway through data collection in TTS. Thematic saturation was reached through the interviews, as no new themes emerged and the data became increasingly repetitive.

#### Data analyses

Questionnaire data were descriptively analysed using Microsoft Excel. Participant characteristics are presented using frequencies and percentages. Descriptive analyses were conducted to summarise demographic characteristics and knowledge questionnaire responses overall and by study site (Kupang and TTS), Interview recordings were transcribed verbatim by the first author and deidentified prior to analysis, which was done after several interviews were conducted. Qualitative data were analysed using the codebook thematic analysis approach.^31,32^ The analysis combined deductive and inductive coding. An initial codebook was developed by the first author through repeated reading of the transcripts and informed by the interview guide and relevant literature, while remaining open to new concepts emerging from the data. The preliminary codebook was discussed and refined with co-authors before being applied to the dataset. All transcripts were imported into NVivo (Version 14; Lumivero) to support data organisation and analysis. The refined codebook was applied to all transcripts, with codes added, modified, or merged as analysis progressed. Throughout the analytic process, the authors met regularly to discuss coding decisions, emerging patterns, and theme development, enhancing the credibility and consistency of the analysis.

The broader doctoral study generated nine major codes relating to midwives’ experiences of providing cervical cancer screening services. For the purposes of this paper, analysis focused on two codes, knowledge and training experiences, which were most relevant to the research questions. The knowledge code captured participants’ discussions of their understanding of cervical cancer, cervical screening, HPV prevention, and cancer-related knowledge. This code appeared in 90% (n=22) of all interviews and produced a rich subset of 128 references. Meanwhile, the training code captured VIA training experiences, peer training, self-learning, HPV DNA test training, and the training needs, which appeared in 95% (n=23) of the interviews and produced another rich subset of 123 references. In reporting the findings, participants are identified by study site and training status. Trained midwives were defined as those who had completed government-certified cervical screening training, whereas untrained midwives had not completed such training. Records of the raw data, field notes, transcripts, coding details, and analysis notes are stored in a secure folder on the University of Melbourne server.

#### Ethical approvals

Ethical approvals were granted by the Human Research Ethics Committee at the University of Melbourne in Australia (Reference Number: 2024-29962-61494-4) on January 22, 2025, and the Medical and Health Research Ethics Committee, Universitas Gadjah Mada, Indonesia (KE/FK/1188/EC/2024) on August 02, 2024. Verbal and written informed consent were obtained from all participants before proceeding with the questionnaires and interviews.

#### Researcher reflexivity

The first author is highly familiar with the local health system in NTT due to being from this region and previously working in a PHC in TTS district, which facilitated rapport and open discussions during interviews. Prior to the study, preliminary discussions with senior midwives in Kupang and TTS informed the refinement of the study focus to ensure service delivery experiences would be captured. To minimise potential bias, the first author maintained a reflexive journal throughout data collection and analysis and regularly discussed reflections and interpretations with co-authors. The second author is a female Indonesian demographer, and the third author is a female medical anthropologist with extensive experience in reproductive rights in Indonesia, both of whom are supervisors of the first author and have been closely involved in methods development and data interpretation.

## Results

### Participants characteristics

Participants’ sociodemographic characteristics are presented in Table 1. Over a third were aged between 41 and 50 years. Most were married, with half the sample having 3 to 4 children. Most participants identified as Christian Protestants, followed by Catholics, which is consistent with NTT province being home to a large Christian population. Two-thirds of midwives held a diploma degree in midwifery, a degree obtained by finishing three years of midwifery education to perform basic midwifery care (see^33^), and worked as civil servants. Half of the participants were VIA coordinators, meaning they had a specific responsibility for coordinating VIA screening in their PHCs. Only a third of the participants had ever had a cervical screening themselves.

**Table 1.** Characteristics of participants.

Characteristics	Kupang (n= 7)	TTS (n = 17)	Total (n= 24)
Age range (years old)			
21 – 30	0	4	4
31 – 40	1	5	6
41 – 50	2	7	9
51 – 60	4	1	5
Marital status			
Single	0	2	2
Married	7	14	21
Widowed	0	1	1
Number of children			
0	0	3	3
1 – 2	2	6	8
3 – 4	5	8	13
Religion			
Christian Protestant	5	14	19
Catholic	2	2	4
Islam	0	1	1
Educational level			
Diploma	5	13	18
Advanced diploma/Bachelor degree	1	4	5
Master’s degree	1	0	1
Employment status			
Civil servant	7	11	18
Contract based	0	6	6
Role			
VIA coordinator	4	8	12
Other PHC-based midwife	1	4	5
Auxiliary PHC midwife	2	0	2
Village based midwife	0	5	5
Years working as a midwife			
1 – 10	1	8	9
11 – 20	1	3	4
21 – 30	2	5	7
>30	3	1	4
Ever had cervical screening			
Yes	4	4	8
No	3	13	16
Had received government accredited cervical screening training			
Trained once	5	5	10
Multiple training	1	1	2
Never been trained	1	9	12
Had One-day introduction workshop on provider administered HPV DNA testing			
Yes	4	0	4
No	3	17	20

Below, we discuss findings under two main themes: a) midwives’ technical training experiences, and b) midwives’ knowledge of HPV, cervical cancer, cervical screening, and HPV vaccination. The relationships between training experience and knowledge are also examined.

### Midwives’ technical training experience

Only twelve out of 24 midwives had been formally trained in cervical screening at the time of data collection (Table 1), despite the current government requirement for all midwives to be competent in cervical screening. In this purposive sample, six out of seven participants in Kupang were trained, compared to six out of seventeen in TTS. Kupang, as the province’s capital, offers greater training opportunities due to its more direct link with government infrastructure, unlike the remote area of TTS (see Table 3).

#### Training content

Prevention of reproductive cancers was explicitly included in the most recent core competencies for Indonesian midwives,^34^ as part of midwifery education for an associate degree. For our participants, cervical screening training on the prevention of reproductive cancers was typically delivered in a combined format, including materials on breast and cervical cancer early detection, and was initiated by the Provincial Health Office (PHO). Usually, the PHO would send invitations to health providers from PHCs across the 22 districts of NTT province to attend training in Kupang if funds were available. Participants who completed training recently recalled that the training for cervical cancer early detection focused on two main topics: VIA and early treatment with cryotherapy.

*“Training materials included VIA and cryotherapy, as well as breast cancer screening. On the last training day, we practised at one PHC with real clients*” – Trained midwife, Kupang.

Midwives who had not been formally trained expressed disappointment at the lack of training opportunities provided to them. An untrained midwife in TTS implied her disappointment that she and her colleagues had not been invited to the training in Kupang, unlike midwives from other PHCs.

*“They held the training last year also. I know because one of my friends from PHC [X] attended the VIA training… The invitations for participants were limited. It was in Kupang. Our PHC was not appointed* [said this sentence with a lower tone]” – Untrained Midwives, TTS

During training, participants were provided with a visual aid, called an “*atlas*”, to illustrate cervical disease progression and typical physiological changes (Figure 1). Observations during interviews revealed that three midwives displayed the visual aids provided during training on the wall in the VIA examination room, while three others placed them on their desks during interviews. This observation illustrates how the training translated into practice for midwives who received these visual aids.

**Figure 1.**
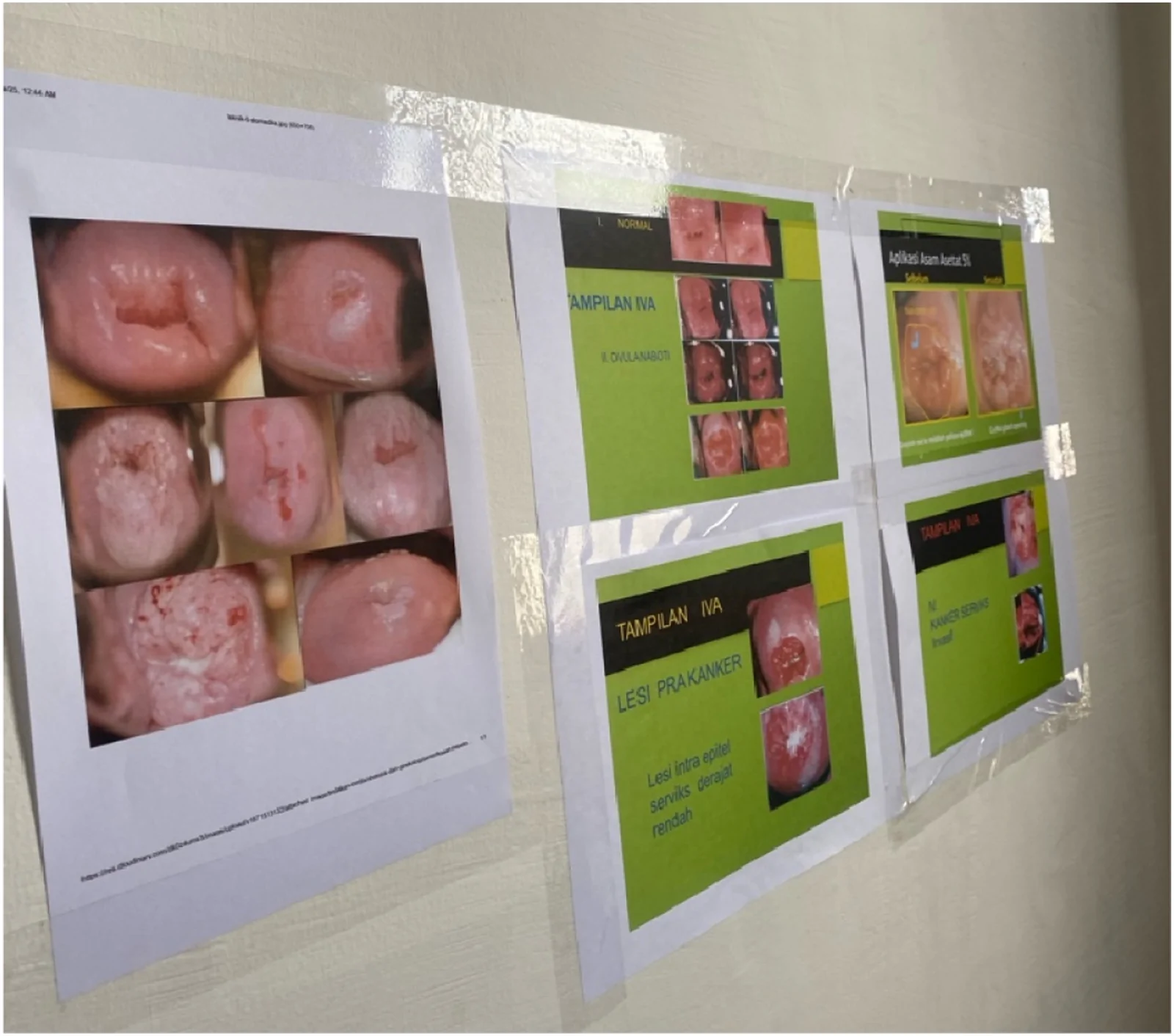
Pictures of the cervix and VIA results were displayed on the wall next to an examination bed at a PHC in TTS (source: documentation during data collection, 25 February 2025).

#### Midwives’ experiences conducting VIA

Trained midwives stated that they felt more confident conducting VIA after being certified as VIA providers. This certification also gave them legal protection, as they asserted: “*[The certificate] is like our driver’s license. Without it, our service would be questioned”* – Trained Midwife, Kupang.

Trained midwives also reported that initially, the process of educating the community about screening and inviting women to screen took more time but became easier the longer they provided the screening information.

*“After we were trained, we did the screening little by little.*.*. It’s like, today we talked about it, but they haven’t decided yet. Tomorrow, we will talk about it again. The more we talked, the more they became interested in screening.”* – Trained Midwife, TTS

#### Unmet training needs and peer training

Four trained senior midwives reported sharing their skills with peers. It was common for midwives in *PHC* to share new knowledge with their peers after completing any training.

*“We usually transmit the knowledge after coming back from any training. What’s it called? Instant training, share a summary of what we learned”* – Trained midwife, Kupang

However, untrained midwives stated that peer training was not enough on its own to make them feel confident in conducting VIA, because they still found it difficult to identify the category of VIA results.

*“We haven't been trained, and we only know what our friend told us. It's also difficult to assess their levels* [VIA results]. *We have a hard time distinguishing between them.”* – Untrained midwife, TTS.

All participants recognised the need for having more trained midwives in their facility or district to offer cervical screening to more women. They agreed that having only one or two trained midwives in a PHC is insufficient. Those who had been trained also asserted the need for refresher courses, as illustrated by the midwife below, who had been provided training over a decade ago.

*“Back then, when I was studying at X [a midwifery school]. I learnt about cervical cancer and the examination. But it was in 2016, a long time ago. Now, for sure I have emm,,, forgotten* [the material]*”* – Untrained midwife, TTS.

Midwives also acknowledged that their knowledge needed updating, particularly in areas such as interpreting VIA results, communication with clients and health promotion. One trained midwife in TTS, who had practised midwifery for less than 10 years, said that she still felt in doubt about pelvic examinations, so she needed to ask senior midwives.

*“I am still in doubt, for like looking for the portio of the cervix. Because if the woman had many children, it would be difficult to find. So, I usually ask the senior midwives to ensure that.”* – Trained midwife, TTS.

None of the trained midwives reported receiving training on health promotion or community engagement to encourage screening uptake, or training on referral pathways for women who test positive. Midwives also emphasised that effective communication skills are crucial for engaging with the community, and that they wanted to enhance their skills in this area.

*“We are especially concerned with how to convey IEC* (Information, Education, Communication)*… To convince the community about this [the need for screening]. I think it requires more skills, because if not, we just talk, they say yes, yes, but no* [people do not come for screening].” – Trained midwife, Kupang.

#### HPV DNA testing training

During the data collection period, the Ministry of Health conducted a pilot project to introduce provider-administered HPV DNA testing in Indonesia, involving Kupang as one of 30 districts nationwide.^35^ The HPV screening training took place in just one day, referred to as ‘on-the-job training’ (OJT) or on-site training, where they visited a PHC and practised HPV DNA testing on real patients. The participants in the HPV training were the VIA coordinators, who had typically received formal VIA training beforehand. While four participants in Kupang attended the on-site training, they reported that there was no comprehensive content on HPV-related topics during the one-day training. Midwives expressed concern about the limited time and knowledge provided in this initial training, as outlined below.

“*It’s not even a training, just an OJT*… *They only gave us materials on how to take samples directly from patients. It’s a pilot project from the Ministry*.” – Trained Midwife, Kupang.

#### Experiences of transition to HPV DNA testing

The HPV DNA testing in Kupang was then conducted concurrently with VIA screening, and this process was referred to as co-testing. Nevertheless, some trained midwives stated that VIA was more convenient because they could see the results immediately. HPV DNA testing in Kupang during the pilot took longer to produce results because samples were sent to the provincial laboratory for analysis, and then the results were sent back to the PHC, a process that could take up to a month.

*“VIA is more practical*. *Because with this* [HPV DNA]*, we have to take a sample, package it, and deliver it. The process has stages and takes a long time. With VIA, we put acetic acid in it, wait a few minutes, and then see the results.”* – Trained Midwife, Kupang.

Midwives’ concern over the time delay related to their ability to re-contact women who might test positive for treatment, which was understood as far less certain if the results took several weeks, than the option of providing women with immediate results and direct referral.

### Midwives’ knowledge of topics related to cervical cancer prevention

Table 2 below includes all knowledge questions, the share of participants with correct answers, and the locations.

**Table 2.** Number of participants with correct answers by each knowledge item and location.

Knowledge statement	Correct answer	Kupang (n=7)	TTS (n=17)	Total (n=24)
**Knowledge on HPV and cervical cancer**				
Human papillomavirus is the main cause of cervical cancer.	True	7; 100%	17;100%	24; 100%
HPV is a sexually transmitted infection (STI).	True	6; 86%	14; 82%	20; 83%
Only women can have HPV infections.	False	6; 86%	3; 18%	9; 38%
Cervical cancer can be treated if detected early	True	7; 100%	17;100%	24; 100%
Only married women over 30 years old can get cervical cancer.	False	6; 86%	11; 65%	17; 71%
Women with cervical cancer sometimes have no symptoms.	True	6; 86%	11; 65%	17; 71%
**Knowledge on cervical screening**				
A positive HPV DNA test means that someone is positively having cervical cancer.	False	1; 14%	1; 0.6%	2; 0.8%
HPV DNA testing is more accurate^1^ than a Pap smear and VIA in identifying women at risk for developing cervical cancer.	True	7; 100%	15; 88%	22; 92%
A negative result of VIA test suggests that a woman will never develop cervical cancer in the future.	False	7; 100%	15; 88%	22; 92%
VIA is a simple and affordable cervical screening method to detect abnormalities in the cervix.	True	5; 71%	16; 94%	21; 88%
The quality of VIA results depends heavily on the provider’s ability to interpret the results.	True	6; 86%	16; 94%	22; 92%
VIA-positives cannot be treated at the same visit with cryotherapy because the client should be referred to an oncologist for further investigation.	False	6; 86%	9; 53%	15; 63%
**Knowledge on HPV Vaccination**				
The best time to receive HPV vaccination is before a person becomes sexually active.	True	4; 57%	14; 82%	18; 75%
Cervical screening is not needed after a person has been fully vaccinated against HPV.	False	4; 57%	13; 76%	17; 71%

^1^The term “accurate” may be ambiguous and should not be interpreted referring to sensitivity and specificity. Here, the statement refers to the ability of HPV DNA testing to identify women at risk of developing cervical cancer.

We also looked at the knowledge scores by participant characteristics (Table 3). None of the midwives in the sample answered all 14 knowledge questions correctly, regardless of their training history. The mean score was 75%, with a range of 8 to 13 correct answers out of 14. Participants from Kupang scored slightly higher than those from TTS, which is likely to be indicative of higher training rates in Kupang. VIA coordinators achieved marginally higher scores compared to other participant groups, reflecting the focus of their roles on CC screening. Those who never performed VIA scored the least compared to the VIA providers.

**Table 3.** Total scores by characteristics.

​	Kupang (n=7)	TTS (n=17)	Total (n=24)
Training status			
Untrained (n=12)	10/14 (72%)	9.5/14 (68%)	9.6/14 (69%)
Trained once (n=10)	11/14 (79%)	11.2/14 (80%)	11.1/14 (79%)
Multiple training (n=2)	13/14 (93%)	12/14 (86%)	12.5/14 (89%)
Roles			
VIA Coordinator (n=12)	11.5/14 (82%)	11.1/14 (79%)	11.3/14 (80%)
PHC Midwives (n=5)	9/14 (64%)	9.8/14 (70%)	9.6/14 (69%)
Auxillary PHC midw (n=2)	11.5/14 (82%)	0	11.5/14 (82%)
Village midwives (n=5)	0	9/14 (64%)	9/14 (64%)
VIA roles			
Main VIA (n=12)	11/14 (79%)	11/14 (79%)	11/14 (79%)
Assisting VIA (n=8)	9.8/14 (70%)	11.5/14 (82%)	10.3/14 (73%)
Not providing VIA (n=4)	0	9.3/14 (66%)	9.3/14 (66%)

#### Knowledge of HPV and cervical cancer

All respondents recognised that HPV is the leading cause of cervical cancer, and it can be treated if detected early (Table 2). However, while twenty participants understood that HPV is an STI, only one-third knew that not only women can contract HPV, with more midwives in TTS answering this item incorrectly. This was reflected in the interviews, with some participants only referring to girls and women as the potential targets for HPV prevention.

*“We should start educating girls from an early age. Then later, when they get married and become mothers, we can remind them again, so they can be more aware.”* – Untrained midwife - Kupang

Midwives’ knowledge scores also reflected a low-risk perception that did not consider all sexually active individuals as being at risk of contracting HPV, and that it was possible for women with cervical cancer to have no visible symptoms. In interviews, the perception that symptoms such as vaginal discharge were symptomatic of cervical cancerwas expressed by ten participants. Many midwives used the presence of vaginal discharge as a starting point to encourage clients to undergo screening.

*“We screen women who come with vaginal discharge symptoms. I have told other midwives to refer to me* [for VIA examination] *if they find cases like that”* – Trained midwife, Kupang

Others described VIA as cervical screening not only for detecting cancer but also for identifying other infections, stating that: *“We explain that VIA was not only for cancer detection, but also for other types of cervical infection”.* – Trained midwife, TTS.

Meanwhile, three untrained midwives were uncertain whether vaginal discharge was related to cervical cancer, *saying, “We do not know if symptoms like vaginal discharge may end up there* [to cancer]?” (Untrained midwife – TTS). Training that clarifies that both HPV and cervical cancer can be asymptomatic is clearly needed to achieve a shift in risk perception so that screening is not only offered to women experiencing vaginal discharge.

#### Knowledge of cervical screening and HPV DNA testing

Only two participants knew that HPV DNA testing is not a diagnostic tool for CC, indicating a low level of understanding of HPV DNA testing (Table 2). From the interviews, we found that twelve midwives in the TTS district and two midwives in Kupang were completely unaware of HPV DNA testing. “*Never heard of it*” or “*Just discovered it when I read the questions (true/false statement)*” were common responses. However, almost all participants answered correctly that HPV DNA testing is the most accurate method for detecting the virus, with all participants in Kupang answering correctly (Table 2). These results appear contradictory but could reflect that participants might have guessed the correct answer based on the term ‘HPV DNA’ being perceived as a new, more advanced technology, because they had not heard of it. However, it is not possible to corroborate this line of thinking.

One midwife from TTS expressed a positive view about the HPV DNA test, mentioning that it could be more effective in detecting the virus.

“[HPV DNA] *is more certain, because it detects the DNA. The virus has many HPV types. So, the test can determine which HPV* [can cause cancer] … *I forgot, was it 14? 12? With HPV DNA, it’s becoming easier to detect*” – Trained midwife, TTS.

Similarly, two participants in Kupang spoke positively about the HPV DNA test, as they understood that its main purpose is to detect the virus.

“*When I'm counselling, I always say the lifespan of the HPV virus is 3-17 years. So, how does it manifest itself in the next maybe 17 years? Even with our own testing, we can be wrong* [refer to VIA interpretation]. *HPV tests are used before symptoms appear, which is really helpful.*” – Trained midwife, Kupang.

As expected, most participants were able to answer questions related to the VIA examination correctly. Only fifteen participants, mostly from Kupang, were aware that theoretically women with a VIA-positive result can be treated during the same visit without confirmation by an oncologist (Table 2). This gap in knowledge is understandable, given that only two out of twelve PHCs in the study sites, all located in Kupang, were equipped to provide cryotherapy services, despite the government’s promotion of a ‘screen-and-treat’ approach.

#### Knowledge of HPV vaccination

The HPV vaccination program for schoolgirls in grades five and six was recently introduced in the study sites, beginning in late 2023. Knowledge about HPV vaccination remained limited among the respondents, with three-quarters of participants understanding that HPV vaccination is most effective and recommended before sexual debut, and the need for ongoing screening after HPV vaccination (Table 2). In the interview, at least six midwives expressed the positivity of HPV vaccines introduced in their community.

“*We started HPV vaccination services in 2023 for girls in grades 5 and 6. This is to protect schoolchildren from the HPV virus.”* – Trained midwife, TTS

A village-based midwife who was under 30 years of age desired to upscale the vaccination also for the young adults, implying midwives' positive attitude toward HPV vaccination.

*“The slogan is 'prevention is better than cure.' I hope this vaccination is expanded not only to schoolchildren but also to adults like me and many others”–* Untrained midwife, TTS

## Discussion

Despite our small sample size, a clear pattern showed that fewer midwives in TTS had received cervical cancer screening training than in Kupang, indicating poorer access to training opportunities for midwives in rural areas. Limited access to training in TTS affected midwives’ knowledge of cervical cancer screening-related topics. The overall mean knowledge score in our study was 75%, which falls within the range reported in other studies for female health workers in African countries, such as in Ghana and Congo.^16,36^ In addition, a systematic review of ten studies assessing female Nigerian health workers’ knowledge of cervical screening reported scores of 54.5 to 90.5%.^37^ To our knowledge, no comparable studies have been conducted in Indonesia. A recent qualitative study assessed Indonesian midwives’ readiness,^20^ highlighting the need to develop a standardised instrument for a national assessment of midwives’ knowledge of HPV and CC screening.

The qualitative findings revealed that the existing training for Indonesian midwives is not comprehensive enough. While participants reported that their training covered the essential VIA components proposed by Brevik^38^– theory-based education, hands-on competency-based skill acquisition, client counselling, visual aids, and competency assessment – the inclusion of breast cancer screening in the training means the depth of content on HPV and cervical cancer cannot be verified. Consequently, the knowledge scores do not indicate the desired levels of knowledge after a focused training program. Improving the comprehensiveness of training on cervical screening has been noted as paramount for increasing the knowledge of para-medical professionals, including midwives, about cervical cancer, HPV, and screening methodology.^14^

Senior trained midwives in this study attempted to address the lack of trained peers in their PHC through spontaneous peer training, but some felt this approach was inadequate. This resonates with other midwifery studies, which indicate that while peer learning is beneficial for collaboration and skill development,^39,40^ it requires careful planning and continuous preparation to meet quality and competency standards.^41^ There was clear demand from senior midwives for a formal train-the-trainer education program that would enable them to provide higher-quality peer training to other midwives, reflecting the strong commitment these midwives expressed towards improving the overall capacity of their practice community in cervical cancer prevention.

### Cervical screening practices among midwives

Only one-third of our participants had a history of cervical screening. We did not ask their reason for not screening, because it would have been culturally disrespectful to challenge midwives on their screening decisions. However, low participation in cervical screening is surprisingly common among female health workers, including nurses and midwives, despite their responsibility to offer cervical screening to communities in LMIC settings, such as in Ethiopia, Nigeria and India.^14,37,42^ Reported reasons for not screening include perceiving it as unnecessary and the service as being inaccessible.^37^ Further investigation into this perception of cervical cancer among Indonesian midwives is needed, including how low-risk perception could be influencing how midwives promote screening to specific client groups and how they conduct health promotion in the community.

We identified several implications of limited knowledge resulting from inadequate training. Midwives’ understanding of HPV infection, as a necessary cause of cervical cancer, is limited; fewer participants were aware that HPV infection can affect all sexes, affecting their understanding of transmission routes via male partners. This lack of knowledge that HPV can be transmitted by male partners was a belief supporting stigma often directed towards women with cervical cancer.^43^ Furthermore, the lack of confidence among untrained midwives in interpreting VIA results emerged as a critical issue, indicating that training is needed to improve provider competence. A study in six countries across Africa and Asia assessed 15 practical training sessions for doctors, nurses, and midwives on cervical screening and early treatment, found that participants’ confidence in delivering the service increased after training, which is consistent with our findings.^44^

Our study documented the early introduction of HPV DNA testing, revealing a stark lack of readiness of the testing infrastructure and of the midwives expected to provide the service. HPV DNA adoption barriers have been observed in other low-resource settings. Studies on the adoption of HPV DNA testing in other LMICs reported that logistical barriers constrained the shift from VIA screening to HPV DNA tests, including associated challenges such as transportation and infrastructure, and human resources hindered the implementation.^7,45^

### Limitations

The limitations of our study include, firstly, that the knowledge questionnaire was intended to provide descriptive and contextual information to complement the qualitative findings, rather than statistically representative estimates. Consequently, no formal sample size calculation was undertaken, and the questionnaire findings cannot be generalised to midwives beyond the study participants. Second, although the questionnaire was developed carefully with reference to cervical screening guidance and the Indonesian context, some items could have been interpreted differently by readers due to variation in country context and prevention strategies. Lastly, although this study generated rich thematic qualitative findings, these are not generalisable to the wider population of Indonesian midwives who work across very diverse communities. The sample was purposively selected from two districts in one outer province in Indonesia, due to familiarity with the area and the feasibility of recruitment despite resource constraints.

## Conclusion

Our findings showed a strong desire among Indonesian midwives for more extensive training in cervical screening and HPV DNA testing, and their knowledge levels confirmed this need. This article also highlights geographical inequality in human resource investment for midwives in rural areas, with more midwives being trained in Kupang than in TTS. We recommend that comprehensive and up-to-date training on cervical screening, HPV DNA testing, and cervical cancer prevention, more broadly, be provided to all midwives in this region to enable them to carry out their tasks effectively. In addition, the transition to HPV DNA testing in Indonesia clearly requires significant investment, especially in improving midwives’ knowledge and training. These efforts are crucial for the successful realisation of the country’s cervical cancer elimination plan.

## Supplemental material

Supplemental material - Exploring Indonesian midwives’ training experiences and knowledge related to cervical screening and HPV DNA testing in East Nusa Tenggara province Supplemental material for Exploring Indonesian midwives’ training experiences and knowledge related to cervical screening and HPV DNA testing in East Nusa Tenggara province by Sandra Olivia Frans, Ariane Utomo, Linda Rae Bennett in Women's Health

## Data Availability

Due to ethical and confidentiality restrictions, the raw data cannot be publicly shared. Deidentified data may be made available upon reasonable request to the corresponding author (SOF) and subject to approval from an Institutional Review Board.*
