# Supplementary material for: Exploring Indonesian midwives’ training experiences and knowledge related to cervical screening and HPV DNA testing in East Nusa Tenggara province
Source: Womens Health (Lond). 2026 Sep 6;22:17455057261487155. doi: 10.1177/17455057261487155 (PMC13735650; doi:10.1177/17455057261487155)
Supplement: Supplementary file 1 [file sj-pdf-1-whe-10.1177_17455057261487155.pdf]

Supplementary File

Table of Contents

Section 1 - COREQ (COnsolidated criteria for REporting Qualitative research) Checklist 2
Section 2 - Interview Guide for Indonesian Midwives 4
Section 3 - Summary of Themes Identified for this paper 5

Section 1 - COREQ (COnsolidated criteria for REporting Qualitative research) Checklist

Topic	Item No.	Guide Questions/Description	Reported on Page No.
**Domain 1: Research team and reflexivity**			
Personal characteristics			
Interviewer/facilitator	1	Which author/s conducted the interview or focus group?	Page 9
Credentials	2	What were the researcher's credentials? E.g. PhD, MD	Page 1,11
Occupation	3	What was their occupation at the time of the study?	Page 1, 11
Gender	4	Was the researcher male or female?	Page 9, 11
Experience and training	5	What experience or training did the researcher have?	Page 11
Relationship with participants			
Relationship established	6	Was a relationship established prior to study commencement?	Page 9, 11
Participant knowledge of the interviewer	7	What did the participants know about the researcher? e.g. personal goals, reasons for doing the research	Page 9, 11
Interviewer characteristics	8	What characteristics were reported about the interviewer/facilitator? e.g. Bias, assumptions, reasons and interests in the research topic	Page 11
**Domain 2: Study design**			
Theoretical framework			
Methodological orientation and Theory	9	What methodological orientation was stated to underpin the study? e.g. grounded theory, discourse analysis, ethnography, phenomenology, content analysis	Page 6
Participant selection			
Sampling	10	How were participants selected? e.g. purposive, convenience, consecutive, snowball	Page 6,7
Method of approach	11	How were participants approached? e.g. face-to-face, telephone, mail, email	Page 7,8
Sample size	12	How many participants were in the study?	Page 8 & 11
Non-participation	13	How many people refused to participate or dropped out? Reasons?	Page 8
Setting			
Setting of data collection	14	Where was the data collected? e.g. home, clinic, workplace	Page 9
Presence of non-participants	15	Was anyone else present besides the participants and researchers?	Page 9
Description of sample	16	What are the important characteristics of the sample? e.g. demographic data, date	Page 12
Data collection			
Interview guide	17	Were questions, prompts, guides provided by the authors? Was it pilot tested?	Page 9
Repeat interviews	18	Were repeat interviews carried out? If yes, how many?	N/A
Audio/visual recording	19	Did the research use audio or visual recording to collect the data?	Page 9
Field notes	20	Were field notes made during and/or after the interview or focus group?	Page 11
Duration	21	What was the duration of the interviews or focus group?	Page 9
Data saturation	22	Was data saturation discussed?	N/A
Transcripts returned	23	Were transcripts returned to participants for comment and/or correction?	N/A

**Domain 3: analysis and findings**			
Data analysis			
Number of data coders	24	How many data coders coded the data?	Page 10
Description of the coding tree	25	Did authors provide a description of the coding tree?	Page 10
Derivation of themes	26	Were themes identified in advance or derived from the data?	Page 10
Software	27	What software, if applicable, was used to manage the data?	Page 10
Participant checking	28	Did participants provide feedback on the findings?	N/A
Reporting			
Quotations presented	29	Were participant quotations presented to illustrate the themes/findings? Was each quotation identified? e.g. participant number	Page 12-21
Data and findings consistent	30	Was there consistency between the data presented and the findings?	Page 12-21
Clarity of major themes	31	Were major themes clearly presented in the findings?	Page 12-21
Clarity of minor themes	32	Is there a description of diverse cases or discussion of minor themes?	Page 12-21

Developed from: Tong A, Sainsbury P, Craig J. Consolidated criteria for reporting qualitative research (COREQ): a 32-item checklist for interviews and focus groups. *International Journal for Quality in Health Care*. 2007. Volume 19, Number 6: pp. 349 – 357

Section 2 - Interview Guide for Indonesian Midwives

**Demographic Information**	
Age range	
Marital status	
Number of children	
Religion	
Education level	
Employment status	
Role at the PHC/health post	
Years working as midwives	
Years working in this PHC/health post	
Ever had cervical screening	
Had received government accredited CC screening training	

**Theme**	**Questions/prompts**
Experience of being midwives	• What motivated you to become a midwife?<br>• Tell me about your experience being a midwife in this Puskesmas (Primary Health care/PHC)?
Sexual and reproductive health (SRH) services and Cervical Cancer perception	• Based on your experience, what SRH services do women most need?<br>• How does the community perceive cervical cancer (CC)? And what do you think influences their perception of CC?
Cervical Cancer prevention policy and program at PHC level	• Are you aware of or have you seen the national CC elimination plan?<br>• In your Puskesmas, do you have a formal CC prevention program?<br>○ If **Yes**, can you tell me what the programs are?<br>○ If **Not**, why do you think Puskesmas does not offer the CC prevention program? Have you discussed any plans to implement the program?<br>• Is CC screening part of the CC prevention program? What techniques do you use for CC screening?
Cervical Screening provision	• Have you ever been trained in the screening technique?<br>○ What is the training about? Tell more about it<br>○ Have you ever received any refreshment training?<br>○ Do you still remember what the contents of the training were?<br>• How do you communicate the need for VIA to women before the test?<br>• What usually happens if you find a VIA-positive case?<br>• Can you conduct cryotherapy in your facility? Please explain
HPV DNA testing	• Does the Puskesmas have HPV-DNA testing?<br>• Who has been trained for HPV-DNA testing in this Puskesmas?<br>• Can you share how the HPV-DNA test works in this Puskesmas?
Barriers and facilitators to CC screening from Midwives' perspective	• What do you consider are the barriers to conducting CC screening?<br>• What support do you need to conduct CC screening?<br>• Have you done dedicated health promotion activities with the community? If yes, how and how often? What are the impact and barriers?<br>○ What did you tell women when you conduct community education about VIA/CC?

Section 3 - Summary of Themes Identified for this paper

Super-ordinate theme	Sub-ordinate themes<br>(number of participants contributing to each theme)
1. Cervical Cancer Knowledge	1. Cervical cancer knowledge or perceptions (20)
	2. HPV infection (15)
	3. HPV DNA test (12)
	4. Cervical screening knowledge (22)
	5. Sexual and reproductive health knowledge (13)
	6. Sexual transmitted infection knowledge (11)
2. CC Training and Update	7. VIA screening training (22)
	8. Peer training (14)
	9. Self-learning (10)
	10. Needs for training (23)
	11. HPV DNA test training (4)
